# Oldie but Goldie: The Fundamental Role of Radioiodine in the Management of Thyroid Cancer

**DOI:** 10.3390/jcm13216400

**Published:** 2024-10-25

**Authors:** Alfredo Campennì, Massimiliano Siracusa, Rosaria Maddalena Ruggeri

**Affiliations:** 1 Unit of Nuclear Medicine, Department of Biomedical and Dental Sciences and Morpho-Functional Imaging, University of Messina, 98125 Messina, Italy; m.siracusadr@alice.it; 2 Endocrinology Unit, Department of Human Pathology of Adulthood and Childhood DETEV, University of Messina, 98125 Messina, Italy; rmruggeri@unime.it

**Keywords:** iodine-131, iodine-123, iodine-124, radioiodine therapy, theragnostic, diagnostic functional imaging, differentiated thyroid cancer

## Abstract

**Background/Objectives**: The management of differentiated thyroid cancer (DTC) patients has undergone a major paradigm shift in past years, especially regarding the role of a careful postoperative disease assessment both in deciding for or against the use of iodine-131 therapy (i.e., patients’ selection) and in selecting the correct goal of the treatment: ablative, adjuvant or therapeutic. Furthermore, diagnostic and risk-oriented uses of iodine isotopes (i.e., ^123/124/131^I) should always be considered during both postoperative assessment and follow-up of DTC patients to improve early staging and response assessment to initial treatments, respectively. The present review summarizes current (and real-life-related) evidence and the emerging perspectives on the therapeutic, diagnostic, and theragnostic use of radioiodine isotopes. **Methods**: A review of the pertinent literature was performed in PubMed, Web of Science, and Scopus without language restrictions or time limits and using one or more fitting search criteria and terms. **Results**: According to the literature evidence and real-life clinical practice, a risk-oriented postoperative iodine-131 therapy remains pivotal for most DTC patients and improves early disease staging through post-therapy functional imaging (i.e., theragnostic aim). Accordingly, the goal of iodine-131 therapy, the optimal strategy (empiric vs. dosimetric approach), the appropriate stimulation method [i.e., levothyroxine (L-T4) withdrawal vs. recombinant human thyrotropin (rhTSH) administration] and, finally, the suggested radioiodine activity to deliver for iodine-131 therapy (RIT) should be personalized, especially in metastatic DTC patients. **Conclusions**: The evidence related to the diagnostic and theragnostic use of iodine isotopes leads to a significant improvement in the postoperative risk stratification and staging of DTC patients in addition to a more accurate assessment of the response to initial treatments. In conclusion, radioiodine is really an *oldie but goldie* radiotracer. It has both a current fundamental role and a future perspective for the more careful management of DTC patients.

## 1. Introduction

The first therapeutic application of iodine-131 was successfully administered for the first time more than 80 years ago by Doctor Saul Hertz to treat a young female patient affected by hyperthyroidism due to Graves’ disease (GD). Since then, millions of patients worldwide have been receiving iodine-131 therapy (RIT) for the definitive treatment of autoimmune (i.e., GD) or non-autoimmune hyperthyroidism [i.e., toxic (multi)-nodular goiter (TMNG/TNG, respectively)], such as through post-surgical therapy of DTC. Iodine-131 decays by β-electrons and emits an electromagnetic photon (i.e., gamma-ray). Based on these physical characteristics, iodine-131 can be considered the first theragnostic agent in nuclear medicine: its gamma-ray emission is used to observe and quantify iodine distribution and kinetics within the target (e.g., thyroid gland or remnant). In contrast, its β-particle emission (β-radiation) produces a therapeutic effect. The physical characteristics of this oldie but goldie radiotracer are useful for the correct clinical, diagnostic, and therapeutic management of DTC patients.

Differentiated thyroid cancer, a follicular cell-originating tumor, is the most frequent thyroid malignancy, and its incidence has been increasing in recent decades with an overall increased annual incidence of about 3% [1,2,3].

The most common DTC histotype is represented by papillary thyroid carcinoma (PTC), accounting for more than 80% of all thyroid malignancies with a significant prevalence of both small tumors and female patients [1,4,5,6,7,8,9].

In DTC patients, the standard of care involves risk-oriented thyroid surgery, postoperative iodine-131 therapy (p-RIT), and both individualized long-term follow-up and levothyroxine therapy tailored according to the patients’ risk of relapse [1,10,11,12,13,14,15]. The goal of postoperative RIT should always be marked as follows: thyroid remnant ablation, adjuvant therapy, and treatment of known metastases.

According to Avram et al. [16], (p-RIT) can be planned by two different empirical approaches: (i) the “functional imaging-guided approach” (i.e., theragnostic approach), based on information obtained with a postoperative diagnostic radioiodine (^123^I, ^131^I, ^124^I) scan, or (ii) the “risk-adapted approach”, based on the local standard of care and the evaluation of clinical–pathological factors other than imaging evidence. Additionally, the dosimetric approach could also be taken into account, at least for patients in whom RIT is performed for ablative or therapeutic purposes.

In addition, RIT represents the primary treatment modality in advanced DTC patients affected by loco-regional and/or distant iodine-avid metastases detected at post-therapy radioiodine scan or during follow-up [10,12,17]. In such patients, radioiodine activity is sometimes preferably chosen by a dosimetric approach using the so-called lesion-based (primarily targeting *efficacy*) or blood-based (primarily targeting *safety*) methods [16,18].

Finally, a risk-adapted use of diagnostic whole-body scintigraphy (Dx-WBS) with radioiodine isotopes (^131^I or ^123^I) (i.e., functional imaging) should always be considered in selected DTC patients both for better assessing the response to initial treatments (i.e., thyroid surgery and RIT) and during late follow-up, as already reported [19,20,21,22,23].

The present review aims to discuss current (and real-life-related) evidence and emerging perspectives on the therapeutic, diagnostic, and theragnostic use of radioiodine isotopes.

## 2. Methods

Starting from the review query, we carried out a literature search of Embase, PubMed/MEDLINE, and Scopus with the purpose of extracting any significant published studies based on the diagnostic, therapeutic, and theragnostic role of radioiodine isotopes. No restrictions regarding either the year of the papers’ release or their language were applied. We used one or more fitting search criteria and terms. The combination terms used for this research are listed below: (A) “Radioiodine therapy” OR “Diagnostic radioiodine” AND (B) “Radioiodine isotopes” or “Radioiodine” AND (C) “differentiated thyroid cancer” OR “thyroid cancer” OR “Radioiodine functional imaging” OR “follow-up” OR “theragnostic”.

## 3. Diagnostic Functional Imaging

The use of diagnostic iodine-131 whole-body scintigraphy (Dx-WBS) has been limited in recent years since some authors argue that it is less sensitive than pT-WBS in detecting metastases other than producing the so-called “stunning effect” of iodine-avid tissue [23,24,25].

However, this criticism should no longer be taken into account for at least two main reasons: (i) technological improvement in nuclear medicine instrumentation [i.e., SPECT/CT imaging (hybrid imaging)], image acquisition and reconstruction protocols, with the resulting possibility to administer lower (≤74 MBq) iodine-131 activity, thus reducing the risk of a stunning effect and increasing the diagnostic performance, especially in terms of sensitivity and specificity, and (ii) the wider availability of iodine isotopes other than iodine-131 (i.e., ^123/124^I), which are able to produce higher-quality imaging, avoiding the stunning effect, and with the consequent further improvement of the diagnostic performance [23,25].

Accordingly, diagnostic functional imaging using different iodine isotopes (e.g., ^131^/^123^/^124^I) can be used during the postoperative assessment of DTC patients to improve their risk stratification and early staging. Thus, the decision-making process can be enhanced in deciding for or against RIT and in choosing its correct purpose (e.g., “functional imaging-guided approach”; see the specific paragraph).

Currently, iodine-131 or iodine-123 Dx-WBS is more frequently used in the follow-up of DTC patients as part of the diagnostic procedures (e.g., laboratory test and nUS) to evaluate the response to initial therapies (e.g., thyroid surgery and RIT) or in DTC patients with recurrent disease [12].

According to the 2015 ATA guidelines, the use of iodine-131 or iodine-123 Dx-WBS at the time of the first follow-up (i.e., response assessment) should be reserved for patients with (1) positive TgAb; (2) poorly informative pT-WBS (e.g., due to the presence of large thyroid remnant); or (3) high-risk DTC [10]. Some authors have also reported on the usefulness of Dx-WBS in patients with metastases at pT-WBS and/or primary malignancy in the isthmus [12,26]. In particular, the isthmus topography of malignant thyroid nodules is an independent risk factor for having both metastatic disease at initial diagnosis and persistent disease at the time of the first follow-up (i.e., response assessment), as already described [26,27,28,29,30,31,32]. Accordingly, in such patients, a more aggressive follow-up, including diagnostic functional imaging, should always be considered [8,26].

Recently, the shared guidelines between the American and European Association of Nuclear Medicine (SNMMI and EANM, respectively) [16] considered important the use of Dx-WBS to (1) establish a new baseline after RIT [33]; (2) determine interval response to RIT; and (3) assess the patient’s thyroid cancer status. Along with laboratory tests (i.e., basal and stimulated-Tg testing) and cross-sectional anatomic imaging, the results of follow-up DxWBS contribute to the dynamic risk restratification of DTC patients.

To date, Dx-WBS (regardless of the iodine isotopes used) should always be integrated by hybrid imaging (i.e., ^123/131^I-Dx-WBS-SPECT/CT) to significantly improve the diagnostic performance of the functional imaging, especially in detecting micro-metastases involving the lymph nodes of the central compartment (i.e., VI Robbins’ level) in patients with negative nUS but a less than excellent response to initial therapies [i.e., bio-chemical indeterminate or biochemical incomplete response (BIndR or BIR, respectively)] [12,34]. In such patients, ^123/131^I-Dx-WBS-SPECT/CT is able to change the response assessment from BIndR/BIR (better prognosis) to structural incomplete response (SIR, worse prognosis), thus significantly modifying their further clinical, diagnostic, and therapeutic management, as recently described [35].

Recently, a basal Tg value ≥ 0.39 ng/mL has been suggested for selecting patients to address ^123^I-Dx-WBS-SPECT/CT to further increase the diagnostic performance of functional imaging and reduce the number of cases that cannot be used [35].

To date, the use of rhTSH (i.e., exogenous stimulation on L-T4 therapy) is supported as the preferred strategy to perform ^123^/^131^I-Dx-WBS-SPECT/CT, which can reduce patients’ discomfort while maintaining the diagnostic accuracy of functional imaging [8,21,22,36].

## 4. Postoperative Iodine-131 Therapy (p-RIT)

The goal of RIT can only be established once the postoperative disease status has been assessed by integrating the histopathological report and the so-called “local factors” (i.e., clinical, laboratory, and imaging parameters, which can be different from patient to patient). Accordingly, the first administration of ^131^I therapeutic activity (i.e., p-RIT) can be used for the following reasons: (1) to destroy normal thyroid tissue remnants in DTC patients classified as low-risk for having persistent/recurrent disease according to the histopathological report (i.e., ablative aim); (2) to irradiate suspected but unproved (e.g., by imaging studies) loco-regional and/or distant metastatic disease in low-to-intermediate or intermediate risk DTC patients (adjuvant aim); or (3) to treat loco-regional and/or distant metastatic disease already noted by imaging studies (therapeutic aim) [16,37].

Noteworthily, p-RIT is used in low-risk DTC patients to simplify their long-term follow-up, which is always necessary in such patients, by using serum thyroglobulin (Tg) monitoring [associated with thyroglobulin antibody (TgAb) measurement] and neck-ultrasound (nUS) (if needed) [38].

**Figure 1:**
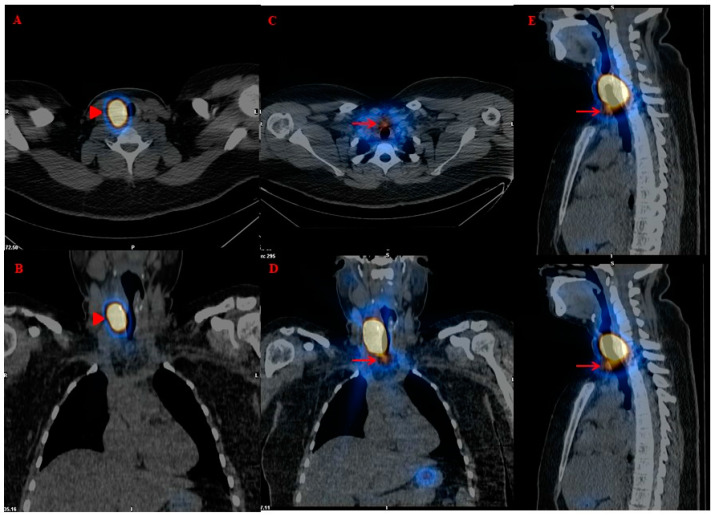
A twenty-six-year-old woman affected by multifocal papillary thyroid carcinoma (12 mm in maximum size), follicular variant, located in the lower part of the left lobe (pT1b(m), Nx, Mx). Postoperative neck-ultrasonography (nUS) performed within three months after thyroid surgery did not show any thyroid remnant and/or suspicious or pathological loco-regional lymph node. The patient underwent rhTSH-aided (standard protocol) iodine-131 therapy (RIT) with ablative purpose (2.2 GBq). At RIT, the peaks of TSH 65 μIU/mL, TgAb 13.6 IU/mL, and basal and stimulated Tg values (day 1, 3, and 5) were <0.04, 3.2, and 10.3 ng/mL, respectively. (**A**,**B**) pT-WBS (anterior and posterior views) and static images (anterior and posterior views) of the neck–thoracic region were obtained two days after RIT. An intense radioiodine uptake consistent with thyroid remnant parenchyma was noted in the right thyroid bed ((**A**,**B**) arrowheads). In addition, a small-sized radioiodine avid lymph node metastasis (red arrows) located in the pre-tracheal region (i.e., VI Robbins’ level) was noted (**C**–**E**).

**Figure 2:**
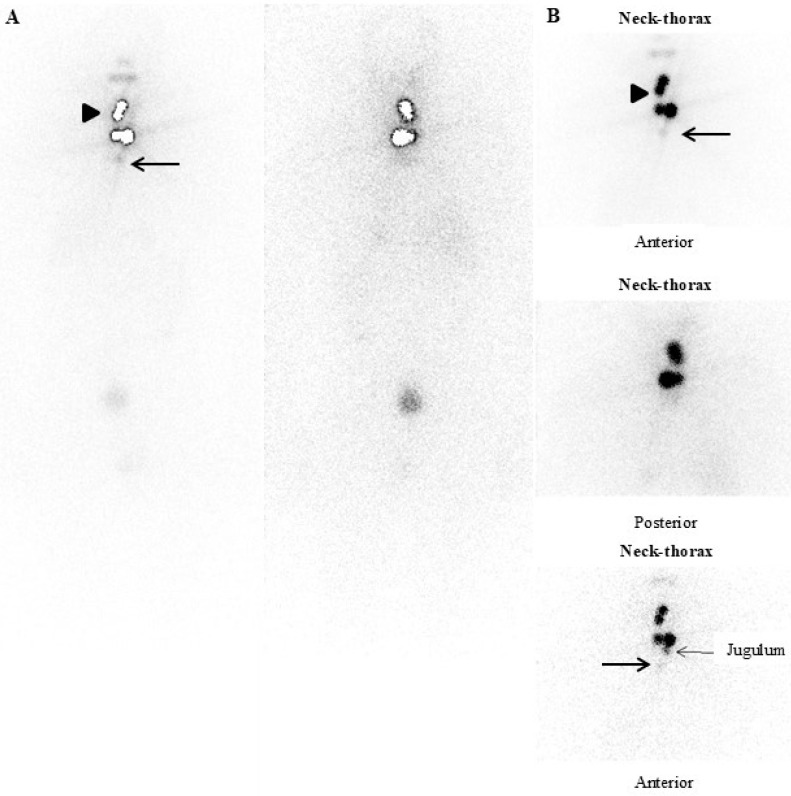
A fifty-two-year-old man affected by papillary thyroid carcinoma (24 mm in maximum size), classical variant, located in the left lobe (pT2, Nx, Mx). Postoperative neck-ultrasonography (nUS) performed within three months after thyroid surgery did not show any thyroid remnant and/or suspicious or pathological loco-regional lymph node. The patient underwent rhTSH-aided (standard protocol) iodine-131 therapy (RIT) with ablative purpose (2.2 GBq). (**A**,**B**): pT-WBS (anterior and posterior views) and static images (anterior and posterior views) of the neck–thoracic region were obtained two days after RIT. Three areas of intense radioiodine uptake (arrowheads) consistent with thyroid remnants were noted in the thyroid bed. In addition, a slight but focal radioiodine uptake consistent with lymph node metastasis was observed immediately below both the thyroid remnant and jugulum region (black arrows). At RIT, the peaks of TSH 111 μIU/mL, TgAb < 10 IU/mL, and basal and stimulated Tg values (days 1, 3, and 5) were always <0.15 ng/mL.

However, in such patients, p-RIT can also be used for an early functional (re)staging based on theragnostic information obtained by ^131^I post-therapy whole-body scintigraphy (pT-WBS), ideally associated with SPECT/CT (Figure 1). Indeed, unexpected loco-regional (i.e., micro-lymph node metastases) and/or distant metastases (more rarely) can be noted in low-risk DTC with undetectable or low Tg levels (in the absence of TgAb) and negative nUS [7,12,39,40,41,42,43,44,45,46,47] (Figure 2). However, false positive radioiodine uptake can be sometimes noted at pT-WBS due to inflammatory or benign disease, as well as some malignant lesions, as already described [48,49]. Noteworthily, the use of hybrid imaging (i.e., SPECT/CT), as a complement to planar imaging, significantly improves the “diagnostic” performance of pT-WBS, reducing the number of either false positive (i.e., radioiodine pitfalls) or false negative results [20,21,36,50].

Although some different points of view exist between endocrinologists and nuclear medicine physicians on the use of p-RIT in lower-risk DTC patients, a strict cooperation within a multidisciplinary team is mandatory for better defined local standards of care. All in all, p-RIT therapy should be considered under the following conditions:

1. Tumor diameter greater than 20 mm: The risk of locoregional and/or distant metastatic disease at diagnosis/initial treatment increases with tumor size, becoming significant for lesions > 2 cm [8,51,52,53,54,55]. The ATA guidelines also report that, in these patients, the risk of structural disease persistence/recurrence (and therefore a more severe prognosis) increases significantly, rising from 1 to 2% in unifocal microcarcinomas to 5% for tumors measuring between 2 and 4 cm [10].
2. Tumor diameter between 10 and 20 mm, multifocal/bilateral: The risk of structural disease persistence/recurrence is not negligible, affecting up to 6% of patients with multifocal and/or bilateral tumors [10,56].
3. Isthmic location: The isthmic location has emerged as an additional and independent risk factor for both the presence of locoregional or distant metastases at diagnosis/initial treatment and disease persistence one year after initial treatments [26,57].
4. Presence of thyroid residue and/or locoregional metastases on postoperative ultrasound and/or functional imaging: Thyroid residue visible on morphological and/or functional imaging (indicating a suboptimal surgery) increases the risk of disease persistence/recurrence, which grows as its size increases, and also limits the diagnostic value of thyroglobulin (Tg) during follow-up. Additionally, the identification of locoregional lymph node metastases on ultrasound imaging indicates radioiodine therapy (with a therapeutic intent) [37].
5. Measurable postoperative basal and/or stimulated thyroglobulin; positive anti-thyroglobulin antibodies: RIT should also be considered for patients with basal and/or stimulated Tg (endogenous or exogenous stimulation using recombinant TSH, rhTSH) above the local institutional cut-off and, in any case, above 2 and 5 ng/mL, respectively [10,16,58,59]. Moreover, RIT should always be considered in patients with positive anti-thyroglobulin antibodies (AbTg). In fact, the positivity of AbTg makes the follow-up of patients more difficult and less reliable, limiting both the diagnostic accuracy and the clinical significance of Tg measurements [10,16,58,59]. The AbTg value is also important since the commonly reported reference cut-offs refer to the adult population with an intact thyroid. Therefore, the use of the Limit of Quantification (LoQ) or the Limit of Detection (LoD) has been proposed to exclude the presence of potentially interfering AbTg [60,61].
6. Presence of locoregional and/or distant metastases on postoperative functional whole-body imaging and “hybrid” imaging (^131-123^I-Dx-imaging): Postoperative diagnostic functional imaging (^123^/^131^I-Dx-imaging) provides information that changes the histopathological risk class in 15% of DTC patients. The use of ^123^/^131^I-Dx-imaging, by revealing the presence of locoregional and/or distant metastases, could modify the clinical management of a significant number of patients, indicating the need for ^131^I therapy with therapeutic intent [16].

In addition to the parameters considered above, the decision for or against the use of RIT for low-to-intermediate risk DTC patients should also take into account local factors that differ among patients according to their demographic, clinical, economic, and social characteristics and provenience. Thus, based on technical–methodological availability, expertise for follow-up, and patient preferences, RIT should always be taken into account when the following cases occur:

- Less than optimal pre- and postoperative neck-US evaluation;
- Less than optimal thyroid surgery (i.e., low-volume thyroid surgeon);
- Poorly informative histological report (i.e., not a standardized report);
- Positive anti-thyroglobulin antibody;
- Limited access to referral centers;
- Patient’s will to maximize the therapeutic process to reduce anxiety related to disease recurrence, considering that the goal of cancer treatment is not only to prolong survival but also to maintain and improve the quality of life.

Finally, ^131^I therapy represents the main treatment option in DTC patients with iodine-avid metastases, and up to 50% of such patients obtain a complete remission or stabilization for a long-term period after RIT, thus having a favorable impact on both overall survival and disease-free survival [62,63,64,65]. The remaining patients are declared to be radioactive iodine refractory (RAI-R) and, as a consequence, RIT is no longer justified [66]. The absence of the sodium iodide symporter (NIS) in the membrane of thyroid cells leads to RAI-R. This is due to gene mutation or rearrangement. Accordingly, the aberrant activation of signal pathways results in the abnormal expression of thyroid-specific genes, leading to RAI-R [67]. In such patients, to date, there are few available and alternative therapies that can be used for the treatment of local (e.g., surgical approach, external beam radiation therapy, interventional radiological procedures) or systemic (e.g., tyrosine kinase inhibitors—TKIs) metastatic disease [12]. The choice of the alternative therapy depends on the loco-regional spread of the tumor as well as the number, size, and location of metastatic disease [12]. Finally, it is important to consider the emerging role of the second-line TKIs (i.e., BRAF and MEK inhibitors) as agents able to restore or enhance radioiodine uptake in RAI-R patients, offering the possibility of a de novo use of RIT [12].

### 4.1. Postoperative Iodine-131 Therapy (p-RIT) Planning (Empiric Approach)

Two empirical approaches can be used to perform p-RIT.

The *“functional imaging-guided approach”* is based on results obtained by postoperative ^131/123^I diagnostic scintigraphy (pDx-WBS) (i.e., theragnostic approach). This approach can significantly improve the postoperative risk stratification and staging of DTC patients by evaluating the topography and extension of iodine-avid thyroid tissue regardless of their initial risk class assigned, according to the 2015 American Thyroid Association [10,16]. In particular, *a functional imaging-guided approach* can be instrumental in the decision-making process of low(er)-risk DTC patients in whom a selective use of RIT is indicated [17,68]. Indeed, a negative pDx-WBS (i.e., no radioiodine uptake or thyroid remnant only) in patients with undetectable Tg value and negative TgAb can lead to either avoiding RIT or reducing radioiodine activity using low instead of moderate approaches. Conversely, a positive pDx-WBS (i.e., unexpected loco-regional and/or distant metastatic disease) corresponds to RIT changing the aim from ablative to therapeutic, thus using higher radioiodine activities that could be personalized by a dosimetric approach [16,69]. In such patients, ^124^I-PET/CT can be considered an alternative functional imaging method to ^131/123^I pDx-WBS due to its favorable characteristics and higher sensitivity in detecting metastases, even if it is more expensive and less readily available [70,71]. In summary, the *“functional imaging-guided approach”* for planning p-RIT is the paradigm of thyroid cancer theragnostics [16,68].

The *“risk-adapted approach”* is based on the accurate evaluation of clinical–pathological factors and institutional protocols [16]. In particular, in patients affected by low- to intermediate-risk DTC, both the decision for or against the use of RIT and the chosen purpose (i.e., ablative or adjuvant) depend on the histological report (accurately evaluated since, to date, a standardized report is not available) and local factors that differ among patients according to their demographic, clinical, economic, and social characteristic and provenience (i.e., rural vs. urban area) [10,12,37]. The histological report and local factors should always be evaluated during postoperative disease assessment, and standardized and integrated into routine clinical care, as already reported [37].

Histological reports must always be accurately evaluated since, to date, a standardized report is not available and a poorly informative one is obtained in a not-negligible number of patients. Among local factors, the quality of pre- and postoperative nUS assessments, quality of Tg measurements (e.g., 1st vs. 2nd generation test), the skills and experience of the thyroid surgeon, and clinical concerns of the local disease management team must be considered in postoperative ^131^I decision making [12,16,72,73,74,75].

Finally (*last but not least*), patients’ will to maximize the intensity of care should always be considered before making a final decision about the pros and cons of using RIT [12,37].

Table 1 summarizes the ^131^I activities suggested according to the risk-adapted approach.

### 4.2. Postoperative Iodine-131 Therapy (p-RIT) Planning (Dosimetry Approach)

The dosimetric approach can be used in DTC patients whose p-RIT is performed for ablative or therapeutic purposes. Conversely, an empiric approach (functional image-guided or risk-adapted) should be preferred if p-RIT is performed with an adjuvant aim, since ^131^I administration is linked to the will to irradiate suspected but unproved metastatic lesion(s) [18,37].

Two dosimetric approaches can be used to perform p-RIT, with ablative or therapeutic aims: (i) the blood or bone marrow dosimetry-based method approach and (ii) the lesion dosimetry-based method [16].

The *blood/bone marrow dosimetry-based method*, primarily targeting safety, is more broadly used, allowing the maximum tolerated activity (MTA) that can be administered to be calculated, and keeping the dose absorbed to the blood/bone marrow (*critical organ*) at ≤2 Gy. In addition, this approach permits preliminary verification that the administered ^131^I activity does not exceed 4.44 GBq in whole-body retention at 48 h, or 3 Gy if pulmonary metastatic disease is present [16,79,80].

Noteworthily, the blood/bone marrow dosimetry-based method permits the avoidance of the risk related to the empiric approach of performing a p-RIT using a radioiodine activity over or under the MTA. Thus, the blood/bone marrow dosimetry-based method is able to avoid overtreatment (which may cause acute dose-related toxicities) [16].

The main limitation of the blood/bone marrow dosimetry-based method is due to the lack of information on the radiation-absorbed dose delivered to the target (i.e., thyroid remnant or loco-regional and/or distant metastases). Accordingly, the method can lead to over- or undertreatment of the target [16].

The goal of the *lesion dosimetry-based method* involves the personalization of ^131^I activity, which would be able to deliver a sufficient radiation-absorbed dose to the target for achieving a therapeutic effect (i.e., ablation of thyroid remnant and/or treatment of metastases) [16]. However, there is no validated method to determine the target mass, either by morphological or functional imaging studies [16,18]. Accordingly, the reported absorbed dose thresholds for providing therapeutic effect change according to different literature data already published. Maxon et al. first proposed a value of 300 Gy to ablate the thyroid remnant and 80 Gy to treat loco-regional metastases. Conversely, Flux et al. proposed 49 Gy for thyroid remnant ablation, while Wierts et al. suggested at least 90 and 40 Gy to deliver treatments for thyroid remnant and loco-regional metastases, respectively [16,81,82,83].

In addition, the target size can be too little to be visualized (e.g., thyroid remnant and/or micro-lymph node metastasis), or metastasis may have loosened its functional ability to take up iodine (i.e., dedifferentiation) [18].

## 5. General Considerations on Patients’ Preparation and Strategy to Deliver Postoperative Iodine-131 Therapy (p-RIT)

In general, a reduction in the daily intake or use of foods [low iodine diet (LID)] and/or products containing iodine should be suggested for two weeks before p-RIT with the aim to optimize ^131^I uptake by normal and/or neoplastic cells (i.e., loco-regional and/or distant metastatic lesions). Similarly, the previous or current use of drugs and/or iodinated contrast media agents must be excluded to rule out an excess of stable iodine [12,84,85,86,87,88].

To date, the measurement of urinary stable iodine excretion is not recommended in all cases. Still, it should be considered in selected patients whose exposure to stable iodine is uncertain [12,89]. In preparation for p-RIT, iodine deprivation is considered adequate or optimal when spot urinary iodine is <100 or 50 µg/L, respectively [16,89,90].

Table 2 summarizes foods, drugs, and iodine-containing substances that reduce radioiodine uptake to thyroid remnants and/or metastatic lesions.

There are two strategies for patients’ preparation for p-RIT: (i) endogenous TSH stimulation by levothyroxine (L-T4) withdrawal (i.e., inducing hypothyroidism) or (ii) exogenous TSH stimulation by administering rhTSH according to the standard protocol (i.e., 0.9 mg/day intramuscularly administered on two consecutive days) [16]. The choice of strategy (i.e., L-T4 withdrawal or rhTSH-stimulation) must be personalized for each patient according to published data [16].

In low-risk DTC patients who underwent p-RIT with an ablative purpose (i.e., thyroid remnant ablation), rhTSH-stimulation proved to be as effective as L-T4 withdrawal. In addition, the use of rhTSH to avoid hypothyroidism can both maintain patients’ quality of life and reduce radiation exposure [12,35,91,92,93,94,95,96]. Consequently, rhTSH-stimulation is the preferred strategy for p-RIT in such patients.

In DTC patients whose p-RIT is performed with an adjuvant purpose (i.e., intermediate-risk DTC), L-T4 withdrawal and rhTSH stimulation should both be considered. In such patients, the choice of stimulation strategy depends on several factors, such as histological features, clinical characteristics, and expected efficacy. In addition, economic feasibility and patients’ quality of life must also be considered [12,16,37,94,97,98,99,100,101,102,103,104,105,106].

Finally, L-T4 withdrawal is currently the preferred strategy for p-RIT (and in case of further treatments) in DTC patients with morphological and/or functional imaging evidence of loco-regional and/or distant metastases. The evidence of iodine-avid metastases at functional imaging can be noted during (a) postoperative assessment according to the so-called *“functional imaging-guided approach”*, (b) at post-therapy whole-body scintigraphy, and (c) during follow-up.

In such patients, the choice of L-T4 withdrawal as the preferred strategy to perform RIT is based on the evidence that metastatic thyroid cells have lower density and poorer functionality of NIS. Accordingly, TSH elevation over time would be important to promote increased ^131^I uptake and retention in tumor cells [10,18,107,108].

Conversely, the literature data recently published indicate that the stimulation strategy (i.e., L-T4 withdrawal vs. rhTSH-stimulation) used to deliver RIT has no significant impact on both RIT efficacy and (as per consequence) metastatic patients’ outcome [12,109,110]. Noteworthily, Gomes-Lima and colleagues did not note any significant difference in terms of progression-free survival and overall survival among fifty-five metastatic DTC patients treated after L-T4 withdrawal (n = 28) or rhTSH-stimulation (n = 27) [109].

Accordingly, the use of L-T4 withdrawal or rhTSH-stimulation as the preferred strategy to deliver RIT in metastatic DTC patients should be deferred to clinical evaluations, taking into account patients’ characteristics and comorbidities [111].

Finally, if a dosimetric approach is chosen instead of an empiric one (e.g., a functional imaging-guided approach or risk-adapted approach), p-RIT (or further treatments) has to be performed with the same functional status (e.g., hypothyroidism or euthyroidism) already used for dosimetric evaluation [12,16].

## 6. Conclusions

Radioiodine isotopes still play a fundamental role in the diagnostic and therapeutic management of most patients affected by benign or differentiated malignant thyroid disorders, representing an evergreen radiotracer family. Currently, the real-life related literature evidence confirms how a risk-orientated and personalized postoperative use of iodine isotopes can heavily impact (re)staging, the subsequent decision-making process (i.e., for or against RIT; the correct and tailored aim of RIT; and therapy modality: empiric vs. dosimetric approach), and the outcome of DTC patients. Additionally, diagnostic functional imaging represents the only diagnostic tool able to detect structural persistent disease in a not-negligible number of DTC patients, thus changing their subsequent assessment and management. Finally, the authors highlight the therapeutic and theragnostic role of iodine-131. It should always be considered as an oldie but goldie chance of definitive care for DTC patients who develop critical status when radioiodine-avid metastases are no longer noted. In conclusion, we wholly support the consideration of radioiodine isotopes as an oldie but goldie diagnostic, therapeutic, and theragnostic tool for the more correct and better-tailored management of patients with thyroid disorders.

## Figures and Tables

**Table 1:** Suggested ^131^I treatment activities in the context of therapeutic intent.

Clinical/Pathologic Context	Prescribed ^131^I Activity	Strategy
Remnant ablation	1.11–1.85 GBq (30–50 mCi) ^131^I *	Risk-adapted ^131^I therapy
Adjuvant treatment	1.85–3.7 GBq (50–100 mCi) ^131^I **	Risk-adapted ^131^I therapy
Treatment of small-volume locoregional disease	3.7–5.6 GBq (100–150 mCi) ^131^I	Risk-adapted ^131^I therapy
Treatment of advanced locoregional disease and/or small-volume distant metastatic disease	5.6–7.4 GBq (150–200 mCi) ^131^I	Risk-adapted ^131^I therapy

* The FDA approved the use of rhTSH in combination with 100 mCi ^131^I for remnant ablation in December 2007 [76,77]. ** Some committee members recommend up to 5.6 GBq (150 mCi) without extant data regarding the effectiveness and toxicity profiles of 1.85 GBq (100 mCi) vs. 5.6 GBq (150 mCi) for adjuvant treatment. Current guidelines advise that the frequency and severity of side effects are activity-dependent, with specifically an increased xerostomia risk for 3.7 GBq (100 mCi) and sialadenitis risk for 5.6 GBq (150 mCi) [78].

**Table 2:** Foods, drugs, and iodide-containing substances that can reduce radioiodine thyroid uptake.

Food and Products That Should Be *Avoided* or *Limited*	Type of Medication	Recommended Time of Withdrawal
Iodized salt **(avoided)**	Water-soluble intravenous radiographic contrast agents	6–8 wk *, assuming normal renal function
Any vitamins or supplements that contain iodine **(avoided)**	Lipophilic intravenous radiographic contrast agents	3–6 mo #
Foods from the sea **(avoided)**	Thyroxine	3–4 wk *
Herbal supplements **(avoided)**	Triiodothyronine	10–14 d ^§^
Eggs **(avoided)**	Methimazole	2–5 d ^§^ before RAI therapy
Milk or other dairy products, including ice cream, cheese, yogurt, and butter **(avoided)**	Propylthiouracil	2–8 wk * if RAI therapy is performed by fixed-activity method (4–7 d ^§^ if RAI therapy is performed after personalized dosimetric approach)
Soy products **(avoided)**	Lugol solution	2–3 wk *, depending on iodide content
Grain products **(limited)**	Saturated solution of potassium iodide	2–3 wk *
Beef, chicken, and turkey **(limited)**	Topical iodine (e.g., surgical skin preparation)	2–3 wk *
	Amiodarone	3–6 mo # or longer

Note: § d, days; * wk, weeks; # mo, months.

## Data Availability

Data available on request.
